# Long-term outcomes of LDR-brachytherapy for localized prostate cancer

**DOI:** 10.3389/fonc.2024.1326355

**Published:** 2025-01-16

**Authors:** Lauri Mäkelä, Anssi Pétas, Arto Mikkola, Harri Visapää

**Affiliations:** ^1^ Comprehensive Cancer Center, University of Helsinki and Helsinki University Hospital, Helsinki, Finland; ^2^ Department of Urology , University of Helsinki and Helsinki University Hospital, Helsinki, Finland

**Keywords:** prostate cancer, LDR, brachytherapy, radiotherapy, long term outcomes

## Abstract

**Introduction:**

This retrospective study aims to evaluate the long-term efficacy and urinary toxicity of LDR-brachytherapy for localized prostate cancer.

**Materials and methods:**

235 primary prostate cancer patients treated with LDR-brachytherapy and subsequently followed up in our center were included in this study. Biochemical relapse free survival (bRFS), overall survival (OS), and cancer-specific survival (CSS) were evaluated. Additionally, the incidence of late urinary complications was recorded.

**Results:**

Median follow-up time was 11,6 years. 181 patients (77%) were classified as low-risk patients, while 52 patients (22,1%) were intermediate risk. The overall bRFS was 83,8% at 5 years and 72,4% at 10 years. 5- and 10-year OS were 97,8% and 87,8% respectively. There was no statistically significant difference in bRFS or OS between different risk groups. The rate of late urinary complications was 8,9%. Volume of prostate had a statistically significant effect on bRFS, as smaller prostate volumes led to worse bRFS.

**Conclusions:**

This retrospective study shows that LDR brachytherapy is an effective treatment for low- and intermediate risk prostate cancer patients with relatively low but still significant risk of late urinary complications.

## Introduction

Prostate cancer is the second most common cancer in men, with over 1,400,000 new cases worldwide in 2020 (1). There are many treatment options for a localized prostate cancer, including active surveillance, radical prostatectomy, external beam radiotherapy, and brachytherapy (2). The choice between these treatment modalities can be challenging, and requires careful weighing of treatment benefits and potential adverse effects while taking tumor and patient characteristics into account.

The rationale of prostate brachytherapy is that it allows a higher dose of radiation to be applied to the target area while minimizing radiation exposure to the surrounding normal tissues. In particular, low dose rate (LDR)-brachytherapy is a prostate brachytherapy technique where radioactive seeds are implanted permanently in the prostate gland and the dose rate of LDR-brachytherapy is defined as ≤2 Gy/h (3).

During the years 2000-2012, low-dose brachytherapy was used at Helsinki University Hospital for treating mainly low- and intermediate-risk localized prostate cancer. Since 2012, it has been replaced with high-dose rate brachytherapy at our center. Additionally, the treatment focus has shifted towards high-risk patients, with HDR-brachytherapy used mostly in combination with external beam radiotherapy. There are differences between these two brachytherapy modalities, some of which are presented in the article by Zaorsky et al. (4). The benefits of LDR-BT include more favorable scheduling logistics, lower initial capital equipment costs, no need for a shielded room, completion in a single implant, and more robust data from clinical trials. These robust data on LDR-brachytherapy can be illustrated by a plethora of retrospective studies reporting 10-year bRFS-outcomes ranging from 79% to 94% (5–12).

The aim of this retrospective study is to present further evidence of the long-term efficacy and the late urinary toxicity of LDR brachytherapy as a treatment for primary prostate cancer with a median follow-up time of over 10 years.

## Materials and methods

We identified 235 primary prostate cancer patients who were treated with whole gland LDR-brachytherapy using Iodine-125 seeds at our center and were subsequentially followed up in the Helsinki University Hospital area.

Primary endpoint was biochemical relapse-free survival (bRFS), which was defined by the Phoenix criteria (PSA nadir + 2 ng/mL). We excluded cases where PSA elevation was transient, required no treatment intervention, and appeared within 2 years from the brachytherapy. Secondary endpoints were overall survival (OS), cancer-specific survival (CSS), and incidence of late urinary complications. Late urinary complications were defined as events that occurred at least 6 months after the brachytherapy and required surgical intervention. The initial treatment in the case of PSA relapse was also recorded and reported.

Clinical T-group, Gleason score, and pre-treatment PSA-value were collected from the patient charts, and the patients were divided into different risk groups using D’Amico risk classification (13). Since there was only a small number of high-risk patients, intermediate- and high-risk patients were combined as one group in the data analyses.

BRFS and OS were calculated according to the Kaplan–Meier curves. Cox regression analysis was used to identify factors (age, prostate volume, PSA, Gleason score, and D’Amico risk group) associated with bRFS and the incidence of long-term urinary toxicity. Statistical analysis was performed with SPSS software version 25.0.

## Results

Median follow-up time was 11,6 years (0,6-21,5 years). The median age of the patients at the time of the procedure was 62 years (46-82 years). 181 patients (77%) were classified as low-risk patients, whereas 52 patients (22,1%) were intermediate-risk and 2 patients (0,9%) were high-risk under the D’Amico classification (**Table 1**).

**Table 1:** Patient characteristics.

Patient Characteristics (n=235)	
**Age median (range)**	62 (46-82)
Primary Gleason score	
** missing**	2
** ≤6**	195
** 7(3 + 4)**	29
** 7(4 + 3)**	3
** 8**	1
Clinical T-stage	
** missing**	20
** T1-T2a**	213
** T2b**	2
D’Amico risk group	
** low**	181
** intermediate**	52
** high**	2
**Prostate volume median (range)**	34,0 cm3 (15,1-61,6cm3)
**Primary PSA median (range)**	6,1 (1,7-20,4)
ADT	
** yes**	9
** no**	226
Treatment information	
** No of needles median (range)**	24 (16-34)
** No of seeds median (range)**	73 (43-105)

N, number of patients; PSA prostate-spesific antigen; ADT, androgen deprivation therapy.

**Figure 1:**
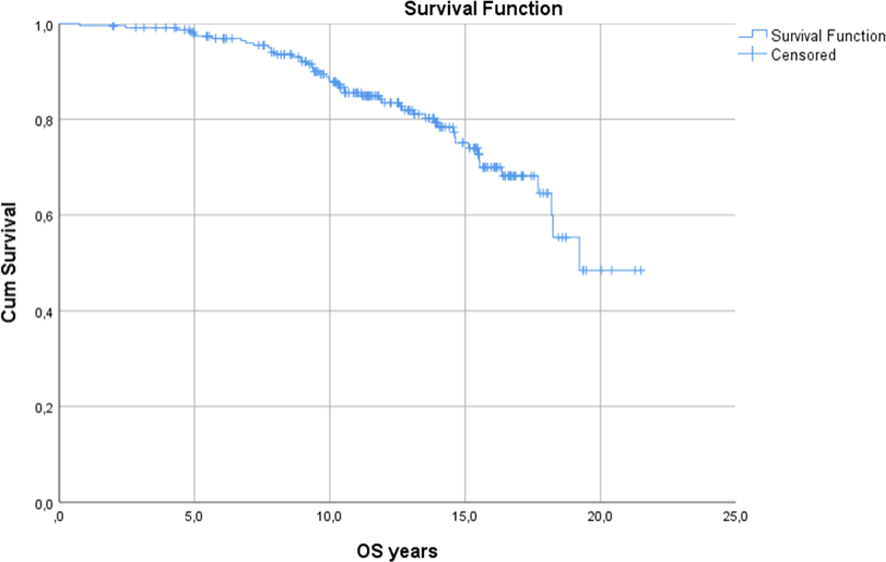
Overall survival.

**Figure 2:**
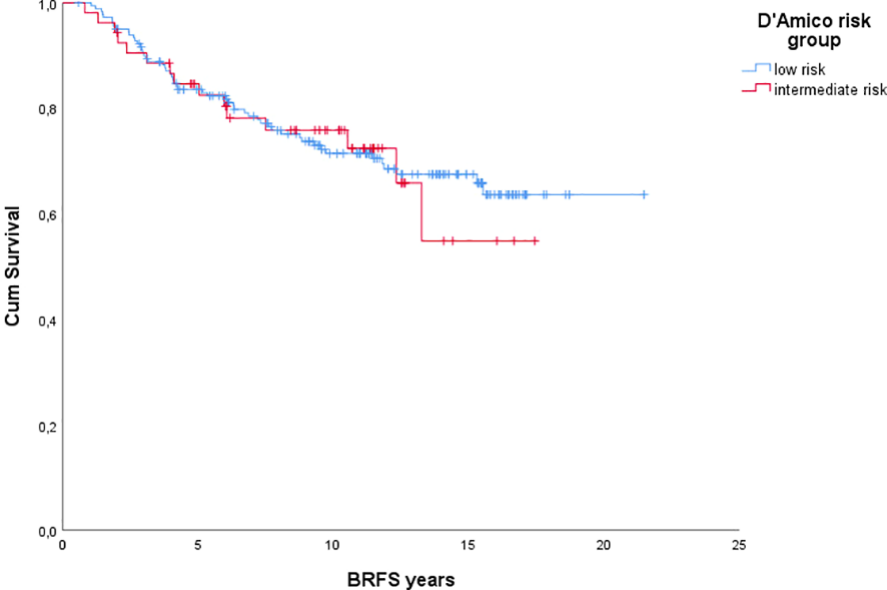
Biochemical relapse-free survival.

The overall bRFS was 83,8% at 5 years and 72,4% at 10 years (**Figure 2**). When stratified by D’Amico risk group, there was no statistically significant difference. The 5- and 10-year bRFS for the low-risk group were 83,6% and 71,4%, respectively. The 5- and 10-year bRFS for the intermediate-risk group were 84,6% and 75,9%, respectively. In both uni- and multivariate analyses, the only variables that affected the bRFS were pre-treatment PSA and volume of prostate (**Table 2**). 5-year overall survival was 97,8% and 10-year overall survival 87,8% (**Figure 1**). Cancer-specific survival was 97% at the end of the follow-up.

**Table 2:** Uni- and multivariate analysis of variables associated with biochemical relapse-free survival.

	Univariate analysis			Multivariate analysis		
	p-value	HR	CI	p-value	HR	CI
Age	0,629	0,99	0,96–1,03	0,460	0,99	0,95–1,03
PSA	0,001	1,16	1,06–1,26	0,000	1,21	1,11–1,32
Gleason	0,225	1,37	0,83–2,27	0,127	1,87	0,84–4,19
D’Amico risk group	0,864	0,95	0,54–1,69	0,054	0,47	0,22–1,01
Prostate volume	0,007	0,96	0,94–0,99	0,001	0,95	0,92–0,98

During the follow-up, 68 patients met the criteria for biochemical relapse. In the event of a biochemical relapse, the most common primary treatment was hormonal treatment (**Table 3**). Five patients were given supplementary LDR-brachytherapy as a deficit was identified in the placement of the seeds in the primary procedure. Median time to the initiation of the treatment from PSA NADIR +2 time point was 217 days. At the end of the follow-up, 15 patients were still on surveillance without treatment intervention despite their serum PSA levels rising over NADIR +2 value. This treatment decision was often made with older patients or when the PSA doubling time was very long.

**Table 3:** Primary treatment in biochemical relapse.

Treatment in relapse (n=68)	
**Hormonal treatment**	35
** Bicalutamide**	32
** LHRH-analogue**	3
**HDR-brachytherapy**	5
**Supplementary LDR-brachytherapy**	5
**Prostatectomy**	8
**EBRT**	1
**Surveillance**	15

N, number of patients; LHRH, luteinizing hormone-releasing hormone; HDR, high dose rate; LDR, low dose rate; EBRT, external beam radiotherapy.

The rate of late urinary complications was 8,9% (n=21). Median time to surgical intervention was 5,3 years (range 0,7-15,1 years). In uni- and multivariate analyses, no statistically significant covariates affected the incidence of these complications. 18 patients received surgical intervention due to urethral stricture. Two patients were surgically treated for urinary incontinence; one patient developed prolonged cystitis symptoms and he was treated with chondroitin sulfate bladder instillation. Two of the 18 stricture patients had been treated with supplementary LDR-brachytherapy. One patient initially developed severe urinary incontinence after supplementary brachytherapy, which ultimately led to a cystoprostatectomy.

## Discussion

The 10-year oncological outcomes in our study are generally comparable to results reported elsewhere. The CSS and OS outcomes presented here can be considered excellent, whereas the bRFS outcomes are a little behind the majority of studies, as 10-year bRFS outcomes in previous studies range from 79% to 94% (5–12).

In the studies reporting better oncological outcomes, there were factors that may have affected the treatment outcome. In our study, adjuvant hormonal treatment was used for only 9 patients and no external beam radiation therapy was given in addition to brachytherapy. As an example, (neo-) adjuvant hormonal treatment was used in most patients in the cohort presented by Morris et al. (9). Furthermore, in the large patient cohort presented by Taira et al. a 12-year bRFS of 95,6% was reported. 49.8% of their patients received supplemental external beam radiation and 37,6% received androgen deprivation therapy (14). Still, the role of adjuvant hormonal treatment with brachytherapy in the low- and intermediate risk-patients is controversial. In a literature review by Keyes et al., 71% of the studies reported a lack of benefit, whereas 28% showed improvement in bPFS with addition of ADT to prostate brachytherapy. It should be noted, however, that only 2 studies included in their analysis were randomized controlled trials (15).

In a study from Kuopio University Hospital, Vuolukka et al. reported the oncological outcome of 241 patients. The relapse-free survival, the cancer-specific survival, and the overall survival were 79,3%, 95,0%, and 66,4%, respectively, with a median follow-up of 11,4 years. They also reported a 10% cumulative incidence of severe urinary toxicity. Their results are in line with the results presented here, with a cohort of similar patient and treatment characteristics, although the use of ADT was slightly more common in their cohort, as 22% received adjuvant hormonal treatment (12).

Furthermore, it is known that after brachytherapy some patients experience a transient elevation in the PSA level, called a bounce. Burchardt and Skowronek report that the median time to PSA bounce was 18 months after LDR brachytherapy (16). In our data, there were seven cases where salvage treatment was started less than 2 years after the brachytherapy. Some of these PSA elevations, which were interpreted as a biochemical relapse, may have been explained by the bounce effect.

There was no statistically significant difference in the bRFS between different d’Amico risk groups. In the EAU-EANM-ESTRO-ESUR-SIOG Guidelines on Prostate Cancer, LDR brachytherapy is recommended for low-risk and intermediate-risk prostate cancer (17). Additionally, in the recent AUA/ASTRO guidelines, LDR brachytherapy can be recommended for low- and favorable intermediate-risk prostate cancer patients (18). Although there was a small proportion of intermediate risk-patients in our study, our results further suggest that LDR brachytherapy is also a viable option for this patient group. In a uni- and multivariate analysis, the only statistically significant variables that affected bRFS were PSA and prostate volume. Interestingly, in our data prostate volume was correlated with bRFS, implying that higher prostate volume led to better bRFS. The correlation was modest, with a hazard ratio of 0,952, but statistically significant. This could be explained by the fact that in a small prostate, urethral sparing may lead to inferior dosimetric coverage of the prostate. To our knowledge there is not much data about the relation between prostate volume and LDR brachytherapy. This was addressed in an article by Chicel et al. regarding HDR brachytherapy in which they concluded that the minimal prostate volume would optimally be ≥ 18 cc to avoid suboptimal treatment plans (19).

The cancer-specific survival at the end of the follow-up was very high at 97%, as only seven patients (3%) died of prostate cancer during the follow-up. Additionally, the 10-year overall survival was high at 87,8%. These results could be attributed to the slow natural course of the low- and intermediate-risk prostate cancer, as well as good therapeutic options in the case of a biochemical relapse. The discrepancy between the bRFS and CSS or OS reported here also adds to the ongoing discussion about the role of bRFS as a surrogate marker for OS. This topic was addressed by the international Intermediate Clinical Endpoints in Cancer of the Prostate (ICECaP) working group, which concluded in their analysis that biochemical-relapse free survival is a weak surrogate end point for OS in localized prostate cancer (20).

These results should be considered in the context of current treatment practices, as most patients in this cohort were low risk, with a Gleason score of 6. For these patients, active surveillance is now the recommended approach (17). There is growing evidence supporting this strategy. Recently, the Canary Prostate Active Surveillance Study (PASS) published results from a prospective cohort of 2,155 patients with favorable-risk prostate cancer managed with active surveillance. 10 years after diagnosis, 49% of patients remained free of progression or treatment, fewer than 2% developed metastatic disease, and less than 1% died from their disease. Although a significant portion of patients initially received treatment, subsequent progression and treatment during surveillance were not associated with worse outcomes (21).

The optimal treatment strategy in the case of a PSA relapse after local curative treatment is often unclear, and differences in patient and tumor characteristics must be considered. In our cohort, the most common treatment choice in case of a biochemical relapse was hormonal treatment, which was initiated in 35 patients (51,5%). Median time from reaching PSA level NADIR+2 to initiation of treatment was 217 days, which was relatively short as these patients were mostly low- and intermediate-risk patients. The initiation of potentially harmful systemic treatment in these patients should be considered carefully. A systematic review investigating the role of hormonal treatment in nonmetastatic prostate cancer recurrence concluded that for most patients hormonal treatment may be more harmful than beneficial, as only patients with aggressive prostate cancer and a rapidly rising prostate-specific antigen might benefit from early hormonal treatment (22).

Late urethral strictures are a known complication of prostate brachytherapy. Historically, the occurrence of surgically treated urinary complications has been shown to fall in the range of 0–8.7% (23). Many studies investigating these complications, however, have relatively short follow-up times. In a meta-analysis by Awad et. Al, the rate of urethral stricture following brachytherapy was 1,9%, but the median follow-up time was only 4 years and an increase in follow-up time was found to increase the risk of developing urethral strictures significantly (p = 0.04) (24). In our study, the rate of urethral strictures was 7,7%, with the median time of incidence at 5,3 years with the last complication recorded 15,1 years after the brachytherapy. Although this rate could still be considered relatively low, it is not insignificant, and should be taken into consideration when making the primary treatment decision, especially in patients with low-risk prostate cancer.

There are some limitations to this study, mostly related to its retrospective nature. Pre-treatment medical history was not assessed and therefore the effect of co-morbidities on oncological outcome and toxicity could not be evaluated. The dosimetric data were incomplete and we were unable to assess the potential impact of dose distribution on oncological outcomes. Additionally, the patient number was relatively small for drawing definitive conclusions about the efficacy of the treatment and especially the factors affecting it. The analysis of long-term urinary toxicity was limited to cases requiring surgical intervention, as this data could be obtained from patient chart reviews. These cases correspond to grade 3 toxicity, effectively bypassing grade 2 toxicities. However, grade 2 toxicities may also include significant long-term effects that impact patients’ quality of life. These were not accounted for in this analysis, leading to an underreporting of meaningful late urinary toxicity outcomes. Moreover, we did not record the incidence of possible secondary cancers which represent a rare but potentially significant long-term risk of prostate radiotherapy (25).

## Conclusions

Here we presented the long-term effects of LDR brachytherapy for low- and intermediate-risk prostate cancer with a long follow-up. Even with this very long follow-up time, the OS and CSS remained high. In addition to showing that LDR brachytherapy is efficient, this could reflect the favorable prognosis of the majority of low- to intermediate-risk prostate cancers. Prostate volume had a small but statistically significant effect on bRFS, and this finding requires further investigation. Furthermore, the incidence of late urinary complications was not insignificant, and needs to be considered when making treatment decisions, especially with low-risk prostate cancer. Further prospective studies are warranted, especially comparing LDR brachytherapy with other treatment modalities, including active surveillance.

## Data Availability

The raw data supporting the conclusions of this article will be made available by the authors, without undue reservation.
